# Impact of Oral Health Education on the Hygiene Awareness of Preschool Children and Their Parents

**DOI:** 10.3290/j.ohpd.c_2274

**Published:** 2025-09-15

**Authors:** Xi Liu, Zheng Wang, Nini Tan, Jinmin Zhao

**Affiliations:** a Xi Liu Researcher, School of Information and Management, Guangxi Medical University, Nanning 530021, Guangxi Province, China; School of Pharmacy, Guangxi Health Science College, Nanning 530023, Guangxi Province, China. Study design and concept, conducting the study, data collation, and writing the manuscript.; b Zheng Wang Researcher, School of Public Health and Health Management, Guangxi Health Science College, Nanning 530023, Guangxi Province, China. Study design and concept, conducting the study, data collation, and writing the manuscript.; c Nini Tan Researcher, School of Pharmacy, Guangxi Health Science College, Nanning 530023, Guangxi Province, China. Study design and concept, conducting the study, data collation, and writing the manuscript.; d Jinmin Zhao Researcher, School of Information and Management, Guangxi Medical University, Nanning 530021, Guangxi Province, China. Study design and concept, conducting the study, data collation, and writing the manuscript.

**Keywords:** oral healthcare, health education, health perception, hygiene awareness, preschoolers

## Abstract

**Purpose:**

The aim of this study was to investigate the impact of oral health education on the hygiene awareness of preschoolers as well as their parents.

**Methods:**

One hundred preschool children who participated in the study were divided into two groups by a randomised numerical table method. The control group (n = 50) received conventional oral health education, while the observation group (n = 50) received simulation education on oral protection. Adverse oral behaviour, toothbrushing and dental health status of children before and after health education, and oral health knowledge acquisition after children’s health education in the two groups are compared. Patients’ hygiene awareness and satisfaction after parental health education were also compared.

**Results:**

The children in the observation group had fewer pacifiers at bedtime, fewer sweet behaviours at bedtime, and fewer lip and finger biting after the health education. Correct brushing posture, use of fluoride toothpaste, brushing time, and number of daily brushing times increased in the observation group after the children’s health education, and all of these were more significant in the observation group. Dental plaque index, gingival index, gingivitis rate, and caries rate were reduced in the observation group of children after health education. The rate of children’s knowledge of oral health education after health education in the observation group was higher than that of the control group in terms of the correct way of brushing teeth, effective prevention of dental caries by correct brushing, bilateral chewing for daily eating, and rinsing the mouth after meals. Oral hygiene awareness and satisfaction with oral health education after parental health education were higher in the observation group than in the control group.

**Conclusion:**

Simulation education on oral protection is beneficial for children to brush their teeth correctly, correct bad oral habits and improve dental health.

**Funding:**

The work was not funded by any funding.

**Conflicts of interest:**

The authors declared that they have no conflicts of interest regarding this work.

Oral diseases are very common, including tooth decay, gingivitis, periodontal disease, and temporomandibular joint disorders, which affect the lives of most people in society. ^14^ Most people’s compromised oral health is related to poor health-related behaviours and nutritional habits. ^17^ Therefore, proper oral hygiene and regular dental monitoring are crucial for maintaining oral health. ^14^ Childhood is a period of high prevalence of dental caries and other dental diseases, as well as a critical period for the formation of oral health behaviours. ^6^ Children with early childhood caries have significantly poorer oral health-related quality of life. Although milk teeth are eventually lost, the disease process continues in the mouth, meaning that dental disease early in life can have lifelong consequences. ^8^

A recent study has shown an association between the parents’ oral health-related quality of life and children’s oral health-related quality of life, indicating that protecting oral health is not just a unilateral goal for children; parents also need to be involved in it. Oral diseases can have a negative impact on the overall growth and development of children, as well as their academic performance and learning abilities. Moreover, parents can also be indirectly affected, as treating children’s oral diseases can lead to family stress, sleep interruptions, economic losses, and other issues. ^24^ Preschool children (children between the ages of 3 and 6 who have not yet reached school age) are in a period of rapid psychological and physical development. Children at this stage have a strong ability to imitate and learn, and are extremely susceptible to bad habits and behaviours, the environment, and other influences. Notably, parental influences on children’s oral health knowledge, attitudes, and behaviours are also significant. ^6^ Parents’ attitudes and knowledge about their children’s oral health could be higher. By modelling healthy behaviours for their children, parents can have a significant impact on the development of healthy oral habits. ^10^ However, while many parents and children have a generally positive attitude towards preventive oral healthcare, there is confusion about how to brush properly, the use of fluoride, and sugar intake. ^16^

The importance of the role of healthcare professionals in children’s oral health has been discussed. ^7^ Recently, the dental team has emerged as a critical workforce for preventive support for parents with young children. The formation of teams of dental providers and family units to adopt protective oral health behaviours is critical to the development of long-term good oral health habits. ^4^ Through effective health conversations between dental hospital staff and parents and children, children are supported in developing optimal oral health habits that align with their daily lives. ^5^ Based on the above, our study focused on the impact of oral health education on the hygiene awareness of preschool children and parents.

## MATERIALS AND METHODS

### Ethical Statement

The study was approved by the Ethics Committee of our hospital. The child’s family signed the written informed consent form.

### General Characteristics

One hundred preschool children participating in a study from June 2022 to May 2023 at our hospital were divided into two groups: a control group of 50 cases and an observation group of 50 cases, using the random number table method. The difference between the two groups of children and their parents was not significant (P >0.05) and comparable (Table 1).

**Table 1:** Comparison of general data between two groups [n(%)]

Indicator	Observation group (n = 50)	Control group (n = 50)	χ²	P
Children’s age			3.144	0.370
3 years old~	4 (8.00)	7 (14.00)	–	–
4 years old~	14 (28.00)	8 (16.00)	–	–
5 years old~	22 (44.00)	21 (42.00)	–	–
6 years old~	10 (20.00)	14 (28.00)	–	–
Children’s gender				0.161	0.688
Male	26 (52.00)	28 (56.00)	–	–
Female	24 (48.00)	22 (44.00)	–	–
Parents’ education				1.026	0.599
Junior/senior high school	31 (62.00)	26 (52.00)	–	–
College/Undergraduate	16 (32.00)	20 (40.00)	–	–
Master’s degree or above	3 (6.00)	4 (8.00)	–	–
Family cohabitants				1.478	0.224
≤ 3 people	24 (48.00)	18 (36.00)	–	–
≥ 4 people	26 (52.00)	32 (64.00)	–	–
Monthly family income					5.938	0.115
<6000 CNY	6 (12.00)	9 (18.00)	–	–
6000 CNY~	27 (54.00)	12 (24.00)	–	–
12,000 CNY~	17 (34.00)	26 (52.00)	–	–
≥ 20,000 CNY	6 (12.00)	3 (6.00)	–	–


### Inclusion and Exclusion Criteria

Inclusion criteria: (1) All preschoolers aged 3 to 6 years old; (2) those who have never been to the dentist before; (3) those who have not had any previous dental related treatment; (4) those who did not receive any oral health education in the recent past; (5) the child’s family was aware of the study and signed a consent form; (6) those who undergone hospital ethical review and signed an informed consent form.

Exclusion criteria: (1) Children or families with psychiatric, psychological, communication or audio-visual impairments that prevent them from cooperating with the study; (2) those unable to provide complete information; (3) those whose age did not meet the relevant requirements; (4) Children who lost visits or dislodged during the study period due to various factors.

## METHODS

Children and their parents in the control group received conventional oral health education: The main content of health education was based on the ‘Oral Health Guidelines for Chinese Residents’ to explain the relevant oral health knowledge, and to explain the correct way of brushing teeth and oral cleaning for children and their families.

Children and their parents in the observation group received simulation education on oral protection: (1) A medical team related to oral health education, consisting mainly of attending physicians and nurses from the dental department of our hospital, was set up. The attending physician acted as the group leader and trained the group members on oral health-related knowledge, requiring all group members to master the correct way of brushing teeth, oral cleaning, care, and the hazards of bad behavioural habits on oral health and other health knowledge content. (2) Before the child visited a doctor, nurses established a WeChat group for oral health education, met with the child’s family face-to-face, and informed them of the role of the group, mainly promoting knowledge related to oral health education. Children and their parents were guided to view the hospital’s own illustrated book on oral health education, which was explained in detail and vividly to the children and their parents. After the children had seen the illustrated picture book, they and their parents were guided to adopt the correct method of brushing their teeth. (3) Nurses pushed a scenario drama on protecting teeth independently developed by our hospital in the group, using their fingers to portray different characters. Through vivid plot, they explained to children the causes and processes of tooth decay, as well as the correct way to brush teeth. (4) The nurses shared a catalogue of picture books related to dental care in the group, including picture books, audio picture books, etc., and encouraged family members to read together with children. By communicating with parents to learn about children’s favourite animated characters, it was easier to regularly promote dental protection-themed animations in WeChat groups and inform parents to watch them together with their children, but the viewing time should be controlled within 2–3 minutes. When the viewing was over, the patients encouraged children to retell the theme content of the animation, and rewarded those who answered actively and correctly. (5) Nurses suggested that parents download some nursery rhymes about tooth protection based on the growth and psychological characteristics of preschool children, and encourage parents to rehearse singing with their children. Nurses recommended some interesting oral health education games and instructed parents to participate in the games. For the game ‘I Love Brushing Teeth’, which lasts 3 to 5 minutes, children only need to move the character’s toothbrush to send it into the mouth to remove bacteria to pass the level successfully. A follow-up survey of research outcomes was conducted in both groups after 12 months of oral health education.

### Observation Indicators

Improvement of adverse oral behaviours before and after health education was compared in both groups of children, including pacifiers at bedtime, bedtime sweetening behaviours, lip licking, and finger biting.

Toothbrushing before and after health education was compared between the two groups of children, including the correct brushing posture, whether or not fluoride toothpaste was used, the time of brushing, and the number of times toothbrushing was performed per day.

Changes in the dental health status before and after health education were compared between the two groups of children, including plaque index, gingival index, gingivitis rate, and caries rate.

The mastery of oral health education knowledge between two groups of children after health education was compared, including the correct way to brush teeth, effective prevention of dental caries through proper brushing, bilateral chewing for daily eating, and rinsing the mouth after meals.

The oral hygiene perceptions of the two groups after parental health education were compared using an assessment document developed by our hospital, which consisted of two aspects: positive attitudes towards oral hygiene (six entries) and correct knowledge of oral hygiene (eight entries).

At the end of the study, the researchers conducted an anonymous survey on the satisfaction level of the two groups of parents with oral health education, using a homemade ‘Satisfaction Survey Instrument Card’ with four levels, ie, Extremely satisfied, Satisfied, Average, and Dissatisfied. The total percentage of ‘Extremely satisfied + Satisfied’ was calculated as the satisfaction level.

### Statistical Analysis

Statistical analysis was performed using SPSS 26.0 software. Qualitative data were described by [n (%)], and a χ² test was performed. Normal distribution quantitative data were described by x– ± s, and independent or paired sample t-tests were performed, and skewed distribution quantitative data were described by M (p25, p75), and Mann–Whitney U tests were performed. P <0.05 was considered a statistically significant difference.

## RESULTS

### Children’s Adverse Oral Behaviours

The differences in all of the adverse oral behaviours of the children in both groups before health education and the differences in all of the adverse oral behaviours of the children in the control group before and after health education were not significant (P <0.05). Compared with the pre-education period, the children in the observation group had fewer pacifiers at bedtime, fewer sweet behaviours at bedtime, and fewer lip and finger biting after the health education (P <0.05). However, the difference between the two groups was not significant after the health education (P >0.05) (Table 2).

**Table 2:** Comparison of children’s adverse oral behaviours between the two groups [n(%)]

Adverse oral behaviors	Observation group (n = 50)	χ²	P	Control group (n = 50)	χ²	P
Before education	After education	Before education	After education
Pacifier at bedtime	4 (8.00)	0 (0.00)	4.167	0.041	5 (10.00)	1 (2.00)	2.837	0.092
Bedtime sweetening behaviour	9 (18.00)	2 (4.00)	5.005	0.025	10 (20.00)	4 (8.00)	2.99	0.084
Lip licking	7 (14.00)	1 (2.00)	4.891	0.027	8 (16.00)	5 (10.00)	0.796	0.372
Finger biting	7 (14.00)	0 (0.00)	7.527	0.006	6 (12.00)	3 (6.00)	1.099	0.295


### Toothbrushing

All the differences in toothbrushing before health education between the two groups of children were not significant, and the differences in toothbrushing before and after health education in the control group were also not significant (P <0.05). Correct brushing posture, use of fluoride toothpaste, brushing time, and number of times per day increased in the observation group after the children’s health education, and all of them were more significant in the observation group (Table 3).

**Table 3:** Comparison of tooth brushing in two groups of children

Toothbrushing	Observation group (n = 50)	χ²/Z	P	Control group (n = 50)	χ²/Z	P
Before education	After education	Before education	After education
Correct toothbrushing posture (n)	20 (40.00)	37 (74.00)^a^	11.791	0.001	18 (36.00)	26 (52.00)	2.597	0.107
Use of fluoride toothpaste (n)	21 (42.00)	46 (92.00)^a^	28.268	<0.001	22 (44.00)	31 (62.00)	3.252	0.071
Brushing time (min)	1.00 (1.00, 2.00)	2.00 (2.00, 2.00)^a^	–5.181	<0.001	2.00 (1.00, 2.00)	2.00 (1.00, 2.00)	–0.968	0.333
Daily toothbrushing (times)	1.00 (1.00, 2.00)	2.00 (2.00, 2.00)^a^	–5.679	<0.001	1.00 (1.00, 2.00)	2.00 (1.00, 2.00)	–1.949	0.051
Note: ^a^P <0.05 vs the control group before education.

### Children’s Dental Health Status

There was no significant difference in the dental health status of the children in the two groups before health education, nor was there a significant difference in the dental health status of the control group before and after health education (P <0.05). After education, the plaque index, the gingival index, the gingivitis rate, and the dental caries rate decreased in the observation group of children after health education. However, the difference between the two groups was not significant after the health education (P >0.05) (Table 4).

**Table 4:** Comparison of children’s dental health status between the two groups

Dental health status	Observation group (n = 50)	Z/χ²	P	Control group (n = 50)	Z/χ²	P
Before education	after education	Before education	after education
Plaque index (points)	1.00 (0.75, 2.00)	0.00 (0.00, 1.00)	–3.921	<0.001	1.00 (1.00, 1.00)	1.00 (0.00, 1.00)	–1.685	0.092
Gingival index (score)	0.00 (0.00, 0.00)	0.00 (0.00, 0.00)	–2.452	0.014	0.00 (0.00, 0.00)	0.00 (0.00, 0.00)	–0.703	0.482
Gingivitis rate (n)	8 (16.00)	1 (2.00)	5.983	0.014	7 (14.00)	5 (10.00)	0.379	0.538
Dental caries (n)	12 (24.00)	2 (4.00)	8.306	0.004	11 (22.00)	7 (14.00)	1.084	0.298


### Children’s Oral Health Education Knowledge

Children in the observation group had a higher rate of mastery of oral health education knowledge after health education than the control group, such as the correct way of brushing teeth, effective prevention of dental caries by correct brushing, bilateral chewing for daily eating, and rinsing the mouth after meals (P <0.05) (Table 5).

**Table 5:** Comparison of children’s oral health education knowledge between the two groups [n (%)]

Oral knowledge	Observation group (n = 50)	Control group (n = 50)	χ²	P
Brushing teeth correctly	37 (74.00)	26 (52.00)	5.191	0.023
Brushing teeth correctly can effectively prevent tooth decay	44 (88.00)	35 (70.00)	4.882	0.027
Bilateral chewing for daily eating	32 (64.00)	20 (40.00)	5.769	0.016
Rinsing mouth after meals	35 (70.00)	21 (42.00)	7.955	0.005


### Parents’ Knowledge of Oral Hygiene

The oral hygiene awareness of the parents in the observation group was slightly higher than that of the control group after health education, which was mainly reflected in the fact that the parents in the observation group agreed more than the control group on the following: ‘Regular oral checkups are essential’, ‘A mother’s poor teeth can affect the child’s teeth’, ‘Bad milk teeth need to be treated’, and ‘Fluoride is useful in protecting teeth’ were higher than those of the control group (P <0.05) (Table 6).

**Table 6:** Comparison of parents’ knowledge of oral hygiene in the two groups [n (%)]

Entry	Observation group (n = 50)	Control group (n = 50)	χ²	P
1. Positive attitude towards oral hygiene:				
(1) Oral health is important to life.	50 (100.00)	50 (100.00)	–	–
(2) Regular oral checkups are essential.	49 (98.00)	43 (86.00)	4.891	0.027
(3) The quality of teeth is not innate and is closely related to self–protection.	50 (100.00)	50 (100.00)	–	–
(4) Preventing dental diseases first depends on yourself.	50 (100.00)	50 (100.00)	–	–
(5) It is important to protect your child’s six-age teeth.	50 (100.00)	47 (94.00)	3.093	0.079
(6) A mother’s poor teeth can affect a child’s teeth.	48 (96.00)	39 (78.00)	7.162	0.007
2. Correct knowledge of oral hygiene:				
(1) Bleeding gums while brushing is not normal.	49 (98.00)	48 (96.00)	0.344	0.558
(2) Bacteria can cause inflammation of the gums.	50 (100.00)	50 (100.00)	–	–
(3) Brushing is useful in preventing bleeding gums.	50 (100.00)	48 (96.00)	2.041	0.153
(4) Dental caries can be caused by bacteria on the tooth surface.	50 (100.00)	49 (98.00)	1.01	0.315
(5) Sugar consumption can lead to dental caries.	50 (100.00)	50 (100.00)	–	–
(6) Bad milk teeth need to be treated.	48 (96.00)	42 (84.00)	4	0.046
(7) Groove sealing can prevent dental caries in children.	41 (82.00)	33 (66.00)	3.326	0.068
(8) Fluoride is useful in protecting teeth.	50 (100.00)	45 (90.00)	5.263	0.022


### Parents’ Satisfaction Levels

Parents’ satisfaction with oral health education was 96.00% (48/50) higher in the observation group than in the control group, 84.00% (41/50) (P <0.05) (Table 7).

**Table 7:** Comparison of parents’ satisfaction levels in the two groups [n (%)]

Satisfaction level	Observation group (n = 50)	Control group (n = 50)	χ²	P
Extremely satisfied	37 (74.00)	29 (58.00)	–	–
Satisfied	11 (22.00)	12 (24.00)	–	–
Average	2 (4.00)	8 (16.00)	–	–
Dissatisfied	0 (0.00)	1 (2.00)	–	–
Satisfaction	48 (96.00)	41 (84.00)	5.005	0.025


## DISCUSSION

Oral health is an integral part of overall health and a significant factor influencing overall quality of life. Despite significant efforts to protect oral health, oral diseases continue to increase. ^9^ Oral diseases are the most common preventable illnesses among children worldwide, and can have a significant impact on the daily lives of children and their families. In addition, oral diseases may adversely affect a child’s speech development, general health and mental health. ^7^ In addition to impacting comfort, function, and ultimately quality of life, poor oral health may affect a child’s physical, cognitive, and psychosocial development. ^22^ Therefore, this study aimed to research the impact of oral health education on the hygiene awareness of preschool children and parents.

There is evidence to suggest that poor oral health in young children is related to their parents’ awareness and behaviour towards oral health. The gum health status between children and parents is highly consistent, which supports the family and behavioural connections established in previous studies. ^19^ Young children are dependent on their parents for healthy behaviours, daily hygiene, habits, and oral health maintenance, including brushing, eating behaviours, and dental visits. ^20^ Providing oral health education to parents to improve their children’s oral health is effective. ^21^ A systematic review emphasised that parents can significantly impact their children’s oral habits by modelling healthy behaviours and maintaining proper oral hygiene themselves. ^10^ However, traditional conventional oral health education is dominated by schools. Since the school is an important place for children to learn and develop, it is natural to use the school environment to teach children about oral health; parents, on the other hand, usually receive simple verbal instructions from dentists in clinics, which have little effect in conveying prevention messages. ^1^ Integrating comprehensive oral health education programmes that actively involve parents can bridge this gap, fostering a more holistic approach to children’s dental care. ^23^ Research has shown that compared to traditional classroom teaching to promote oral health knowledge, oral health education games stimulate and deepen children’s understanding and desire for oral health knowledge through interactivity and fun. ^13^ These games engage children through interactivity and enjoyment, thereby stimulating and deepening their understanding and interest in oral health. Furthermore, incorporating gamification into oral health education has shown potential as an innovative approach to promote positive health behaviours. A scoping review reported that most studies on gamification in oral healthcare observed improvements in children’s oral hygiene practices, suggesting that such interactive methods can effectively enhance engagement and knowledge retention. ^15^ It is suggested that the integration of interactive, game-based educational strategies, alongside active parental involvement, can significantly enhance oral health outcomes in children by fostering better understanding, engagement, and application of oral hygiene practices.

Our study demonstrated that children who received simulation education on oral protection had fewer pacifiers at bedtime, fewer sweet behaviours at bedtime, and fewer lip and finger biting, which indicated that simulated oral education could effectively reduce children’s poor oral behaviours after the health education. These findings suggest that interactive, experiential learning methods effectively diminish poor oral behaviours among preschoolers. Through simulation, children acquired essential oral hygiene skills, including proper toothbrushing techniques. This aligns with research indicating that experiential learning enhances knowledge and attitudes in health education, particularly when interactive methods are employed. ^3^ One of the interesting insights gained from a study is that children want to be respected, listened to, valued, and truthfully informed, so they can participate in decisions about their care and be accepted and valued in the oral healthcare process. As a consequence, the inclusion of children’s ideas in dental teams and family-based formation groups can be effective in improving their oral health awareness. Some studies have proven that edutainment plays a vital role in meeting the healthcare needs of diverse populations. Considering the positive effects of education, it could be said that children and parents can significantly enhance their awareness of teeth and oral hygiene through storytelling and playing games. ^18^ Interactive learning methods, such as storytelling and games, have been shown to significantly enhance oral health knowledge and hygiene practices among children and their parents. For instance, a randomised clinical trial demonstrated that video-based edutainment effectively improved oral health education outcomes in school-age children. ^11^ In our study, parents’ knowledge of oral hygiene and satisfaction levels were both enhanced after education, showing that simulation education on oral protection has more advantages than conventional oral health education. Research supports that family involvement in oral health education significantly influences children’s oral health behaviours and outcomes. ^12^ Collectively, implementing interactive, simulation-based oral health education strategies positively influences the oral hygiene awareness and behaviours of preschool children and their parents.

## CONCLUSION

In summary, this study demonstrated that oral health education has a positive and essential impact on improving the hygiene awareness of preschool children and their parents. Specifically, the intervention effectively reduced poor oral behaviors in preschool children, guided them to develop correct toothbrushing habits, increased both children’s and parents’ knowledge of oral health, and enhanced parents’awareness and satisfaction regarding oral healthcare. These findings are consistent with the study’s aim and highlight the importance of early oral health education in promoting healthy behaviours and awareness among preschool children and their families. However, due to the limited clinical data available, further large-scale, multicentre studies with extended follow-up periods are warranted to comprehensively explore the effectiveness and long-term impact of oral health education.

## References

[ref1] Aleksejuniene J, Pang RHI (2023). Peer-led oral health education model for elementary school-aged children in British Columbia, Canada. Can J Dent Hyg 2022; 56:72–82.Alwadi MAM, Baker SR, Owens J. Oral health experiences and perceptions of children with disabilities in the Kingdom of Saudi Arabia. Int J Paediatr Dent 2022;32:856–864.Angelopoulou MV, Kavvadia K, Taoufik K, Oulis CJ. Comparative clinical study testing the effectiveness of school based oral health education using experiential learning or traditional lecturing in 10 year-old children. BMC Oral Health 2015;15:51.Bhatti A, Gray-Burrows KA, Giles E, Rutter L, Purdy J, Zoltie T, et al. ‘Strong Teeth’: the acceptability of an early-phase feasibility trial of an oral health intervention delivered by dental teams to parents of young children. BMC Oral Health 2021;21:138.Bhatti A, Vinall-Collier K, Duara R, Owen J, Gray-Burrows KA, Day PF. Recommendations for delivering oral health advice: a qualitative supplementary analysis of dental teams, parents’ and children’s experiences. BMC Oral Health 2021;21:210.Chen L, Hong J, Xiong D, Zhang L, Li Y, Huang S, Hua F. Are parents’ education levels associated with either their oral health knowledge or their children’s oral health behaviors? A survey of 8446 families in Wuhan. BMC Oral Health 2020;20:203.El-Yousfi S, Marshman Z, Albers PN, Watt S, Kipping R, Williams JG. Health visiting teams and children’s oral health: a scoping review. BMC Oral Health 2022;22:594.Gussy M, Mnatzaganian G, Dashper S, Carpenter L, Calache H, Mitchell H, et al. Identifying predictors of early childhood caries among Australian children using sequential modelling: findings from the VicGen birth cohort study. J Dent 2020;93:103276.Ivanisevic Z, Uzarevic Z, Lesic S, Vcev A, Matijevic M. Oral health of children from the SOS children’s village in Croatia. Int J Environ Res Public Health 2021;18:616.Kaushik M, Sood S. A systematic review of parents’ knowledge of children’s oral health. Cureus 2023;15:e41485.Lekaram W, Leelataweewud P, Kasemkhun P. Effectiveness of edutainment use in video-based learning on oral health education for school-age children: a randomized clinical trial. BMC Oral Health 2025;25:381.McKinney DC, Sullivan M. The importance of family oral health education in promoting children’s oral health. J Dent Hyg 2024;98:4–5.Mendonca TS, Carvalho ST, Aljafari A, Hosey MT, Costa LR. Oral health education for children: development of a serious game with a user-centered design approach. Games Health J 2024;13:268–277.Minervini G, Franco R, Marrapodi MM, Di Blasio M, Ronsivalle V, Cicciù M. Children oral health and parents education status: a cross sectional study. BMC Oral Health 2023;23:787.Moreira R, Silveira A, Sequeira T, et al. Gamification and oral health in children and adolescents: scoping review. Int J Med Res 2024;13:e35132.Naidu RS, Nunn JH. Oral health knowledge, attitudes and behaviour of parents and caregivers of preschool children: implications for oral health promotion. Oral Health Prev Dent 2020;18:245–252.Shay B, Ben Ami O, Levy Ianculovici D, Zini A, Ianculovici C, Almoznino G. Oral health-related quality of life in patients with disorders of nutrition. J Oral Rehabil 2019;46:355–368.Shruti T, Govindraju HA, Sriranga J. Incorporation of storytelling as a method of oral health education among 3-6-year-old preschool children. Int J Clin Pediatr Dent 2021;14:349–352.Stormon N, Clifford S, Lange K, et al. Oral health: Epidemiology and concordance in Australian children and parents. Community Dent Oral Epidemiol 2022;50:260–269.van Spreuwel P, Jerkovic-Cosic K, van Loveren C, van der Heijden G. Parents’ willingness to invest in primary oral health prevention for their preschool children. Int J Environ Res Public Health 2021;18.Wang K, Yu KF, Liu P, Lee GHM, Wong MCM. Can mHealth promotion for parents help to improve their children’s oral health? A systematic review. J Dent 2022;123:104185.Watt S, Dyer TA, Marshman Z, Jones K. Does poor oral health impact on young children’s development? A rapid review. Br Dent J 2024;237:255–260.Wu K, Yin W, Liang X, Zou L, Yang Z. The influence of parents’ oral health literacy and behavior on oral health of preschool children aged 3-6 years- evidence from China. BMC Oral Health 2024;24:1445.Yang L, Zhao S, Zhu Y, Lai G, Wang J. Oral health-related quality of life and associated factors among a sample from East China with severe early childhood caries: a cross-sectional study. BMC Oral Health.

